# Supplementary material for: Evidence supported by Mendelian randomization: impact on inflammatory factors in knee osteoarthritis
Source: Front Med (Lausanne). 2024 May 28;11:1382836. doi: 10.3389/fmed.2024.1382836 (PMC11165061; doi:10.3389/fmed.2024.1382836)
Supplement: Supplementary file 1 [file Data_Sheet_1.ZIP › data/GCST90274811_5e6/811.forest.pdf]

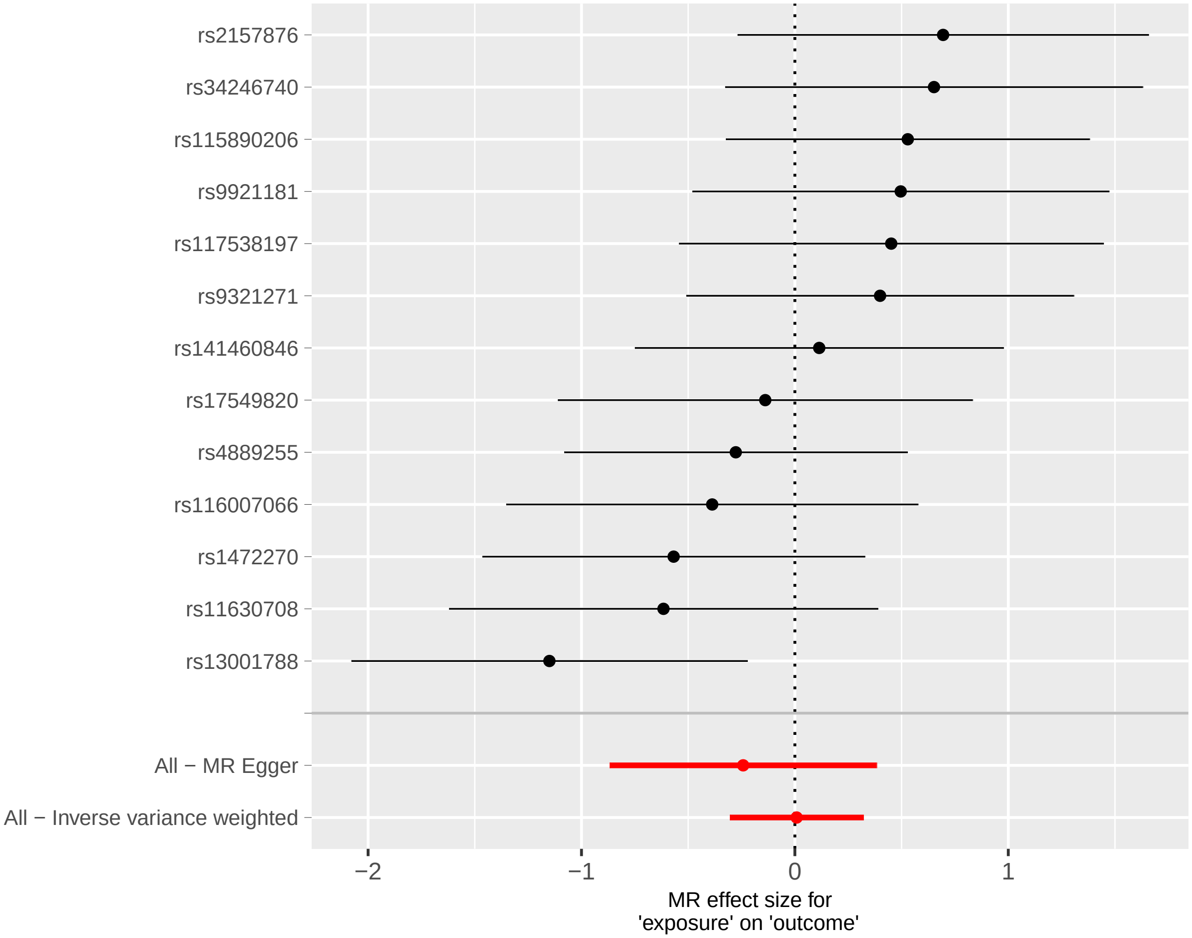
rs2157876
rs34246740
rs115890206
rs9921181
rs117538197
rs9321271
rs141460846
rs17549820
rs4889255
rs116007066
rs1472270
rs11630708
rs13001788
All − MR Egger
All − Inverse variance weighted
−2
−1
0
1
MR effect size for
'exposure' on 'outcome'
